# Age-Associated Metabolomic Changes in Human Spermatozoa

**DOI:** 10.3390/ijms27052386

**Published:** 2026-03-04

**Authors:** Mohd Amin Beg, Md Shawkat Khan, Ishfaq Ahmad Sheikh, Taha Abo-Almagd Abdel-Meguid Hamoda, Mohammad Imran Khan, Priyanka Sharma, Ali Hasan Alkhzaim, Kenaz Basem Abuzenada, Arif Mohammed, Abrar Ahmad, Adel Mohammad Abuzenadah, Erdogan Memili

**Affiliations:** 1 King Fahd Medical Research Center, King Abdulaziz University, Jeddah 21589, Saudi Arabia; mkhan0082@stu.kau.edu.sa (M.S.K.); iasheikh@kau.edu.sa (I.A.S.); aabuzenadah@kau.edu.sa (A.M.A.); 2 Department of Biochemistry, Faculty of Science, King Abdulaziz University, Jeddah 21589, Saudi Arabia; aaldin@kau.edu.sa; 3 Department of Urology, Faculty of Medicine, King Abdulaziz University Hospital, Jeddah 21589, Saudi Arabia; tahaaboalmagd@yahoo.com (T.A.-A.A.-M.H.); ali.ali-14@hotmail.com (A.H.A.); 4 Department of Urology, Minia University, Minia 61519, Egypt; 5 Research Center, King Faisal Specialist Hospital & Research Center, Jeddah 21499, Saudi Arabia; imrankhaniitr@gmail.com; 6 Center of Innovation in Personalized Medicine, King Abdulaziz University, Jeddah 21589, Saudi Arabia; priyanka82jnu@gmail.com (P.S.); kabuzenada@gmail.com (K.B.A.); 7 Department of Biological Sciences, College of Science, University of Jeddah, Jeddah 23890, Saudi Arabia; aarifsls@yahoo.co.in; 8 Research Center, King Faisal University, Hofuf 36363, Saudi Arabia; 9 Cooperative Agricultural Research Center, College of Agriculture and Human Sciences, Prairie View A&M University, Prairie View, TX 77446, USA; ermemili@pvamu.edu

**Keywords:** human, aging, spermatozoa, metabolomics, LC-MS/MS

## Abstract

The functional genomic mechanisms contributing to aging-associated decline in fertility in men remain insufficiently elucidated. This study investigated age-related alterations in the sperm metabolome of healthy fertile Arab men across three groups: young adult (21–30 years, *n* = 6), late adult (31–40 years, *n* = 7), and advanced age (41–51 years, *n* = 5). Metabolomics was performed using LC-MS/MS. Statistical/functional analyses were performed using MetaboAnalyst-Pro. A total of 380 metabolites were identified, of which 164 showed significant differences (*p* < 0.05) across age groups. Principal component analysis, partial least squares-discriminate (PLS-DA), and sparse PLS-DA consistently demonstrated distinct metabolomic clustering between young adult and advanced age groups. Notably, in the advanced-age spermatozoa, L-homocysteine was undetectable, while methyloctadecanoyl-CoA was uniquely abundant. Biomarker analysis identified 137 potential aging-sperm biomarkers (AUC = 1), including upregulated (e.g., pentadecanoyl-CoA, (3S)-3-hydroxylinoleoyl-CoA, CDP-DG(LTE4/20:4(8Z11Z14Z17Z)), uracil) and downregulated (e.g., (S)-hydroxyoctanoyl-CoA, DG(22:6/18:4), L-homocysteine, N-myristoyl serine) metabolites. These biomarkers participate in key sperm domains, including motility, energy metabolism, membrane remodeling, oxidative-stress regulation, and fertilization. In conclusion, advancing age disrupts sperm “metabolostasis” (metabolite homeostasis essential for normal function), compromising their physiological integrity and fertilization competence. The identified biomarkers offer promising targets for interventions to preserve sperm health and mitigate age-related fertility decline.

## 1. Introduction

Aging-associated fertility decline in men is assuming significant global importance, due to evolving lifestyle choices and demographic changes [1,2,3,4,5]. The growing tendency to delay childbearing has led to more advanced-age couples seeking fertility treatment, further exacerbating the issue [6,7,8]. Although maternal age effects are well studied, paternal mechanisms of age-related fertility decline remain poorly understood [5,9,10]. Men typically never stop producing spermatozoa, but age-related sperm structural and functional changes significantly reduce chances of conception [4,11,12]. These changes include declining testosterone, reduced ejaculate volume, lower sperm count and quality, increased DNA fragmentation, and compromised membrane integrity [12,13,14,15,16,17]. Moreover, aging increases the probability of chromosomal abnormalities, genetic mutations, and epigenetic alterations in sperm, adversely affecting embryo development and offspring health [2,9,18]. Furthermore, age-associated comorbidities such as diabetes, cardiovascular disease, and obesity further impair sperm production and quality [19,20,21]. Notably, live birth rates in women aged 35–39 years declined with advancing paternal age, from 27% (<35 years) to 19% (>50 years) [22].

Globally, societal and lifestyle changes and industrialization have led couples to prioritize financial stability, education, and careers, contributing to delayed parenthood [14,23]. In the United States, the average paternal age rose from 27 years in 1972 to 32 years in 2015, with proportion of fathers aged ≥40 and ≥50 doubling from 4.1% to 8.9% and 0.5% to 0.9%, respectively [24]. Between 1980 and 2015, births among men aged 35–49 rose by 60% [25]. Similar demographic trends are projected for Saudi Arabia, driven by industrialization, education, and workforce participation [26,27]. The median age is projected to rise from 29 in 2020 to 40 years by 2050, with 25% of the population 60 years or older [28]. The fertility research in Saudi Arabia remains limited; infertility affects about 19% of couples, often necessitating assisted reproduction [29,30]. Overall, this demographic transition accentuates aging as an emerging public health priority, with genetic, reproductive, and transgenerational implications [23,31,32].

Although paternal aging is increasingly implicated in poor reproductive outcomes, its effects on male fertility remain insufficiently characterized. Aging is linked to impaired spermatogenic support and reproductive dysfunction, marked by reduced Sertoli, Leydig, and germ cell populations [33,34]. Age-related decline in semen quality is also well documented, notably, a decline in semen volume by about 35 years [9,12,34] and significant reductions in sperm count, viability, and motility around 40 years [11,12,35,36,37]. While advancing paternal age is associated with reduced ejaculate quality, little is known about age-related sperm functional genomic alterations, particularly metabolomic changes. The metabolome is a sensitive indicator of global cellular function, and metabolomics profiles low-molecular-weight compounds (sugars, amino acids, lipids, nucleosides, vitamins), offering critical insights into cellular physiological status [38,39,40,41]. In male fertility research, metabolomics delineates clinical phenotypes with underlying pathobiological mechanisms in spermatozoa, identifying metabolites central to motility, metabolism, and fertilization [40,42,43,44,45]. Such analyses are critical for understanding aging-related sperm dysfunction and developing interventions.

Several recent studies have characterized the human sperm metabolome in relation to fertility. An LC-MS/MS study of spermatozoa from fertile men identified 171 metabolites, many correlating with sperm motility [46]. In contrast, asthenozoospermic and other infertile phenotypes consistently showed metabolic disruptions, including elevated saturated and monounsaturated fatty acids and reduced polyunsaturated fatty acids, likely impairing membrane fluidity and motility [47,48,49]. Untargeted GC-MS and HPLC studies further revealed altered amino acids, nucleotides, lipids, and redox-related metabolites in asthenozoospermic spermatozoa, indicating compromised metabolism, antioxidant activity, and genetic functions [50,51]. Comprehensive LC-MS analyses also demonstrated disrupted amino acid, energy, and lipid metabolism in asthenozoospermia and obesity-associated infertility [45,52,53].

The current literature on aging and sperm metabolomics is limited. To our knowledge, no study has examined the age-related metabolomics of normal human spermatozoa. A recent study reported whole semen metabolomics in two age groups [54]. However, participants were fertility clinic attendees, potentially confounding aging effects. Comparing younger (26–30 years) and older men (45–54 years), the study identified significant pathway-specific metabolomic differences and proposed four metabolites as biomarkers of human semen aging.

With rising delayed fatherhood and shifting demographics, understanding age-related alterations in spermatozoa is critical for addressing fertility decline in aging men. The paucity of data underscores the urgent need for rigorous investigations incorporating well-defined age groups and confirmed fertility status to elucidate age-associated sperm metabolomic changes. This novel study analyzed spermatozoa from three reproductively-relevant age groups of fertile men: young adult (21–30 years), late adult (31–40 years), and advanced age (41–51 years). The aims were to characterize differentially abundant sperm metabolites across age groups and identify key metabolite biomarkers, with diagnostic and therapeutic relevance for fertility preservation in aging couples. The findings offer novel insights into sperm metabolomic alterations with advancing paternal age.

## 2. Results

### 2.1. Semen Analysis

The mean data for age and semen endpoints were detailed recently [55] and are summarized in Table 1. Briefly, none of the comparisons for semen endpoints were significant except the age of men and the spermatozoa concentration. The mean age of men was different (*p* < 0.001) among each of the three groups. The mean spermatozoa concentration was lower (*p* < 0.05) in the young adult group compared to the late adult group, but comparisons with the advanced age group were not significant (Table 1).

### 2.2. Spermatozoa Metabolome

#### 2.2.1. Overall Metabolomic Analysis in Three Age Groups

**Figure 1:**
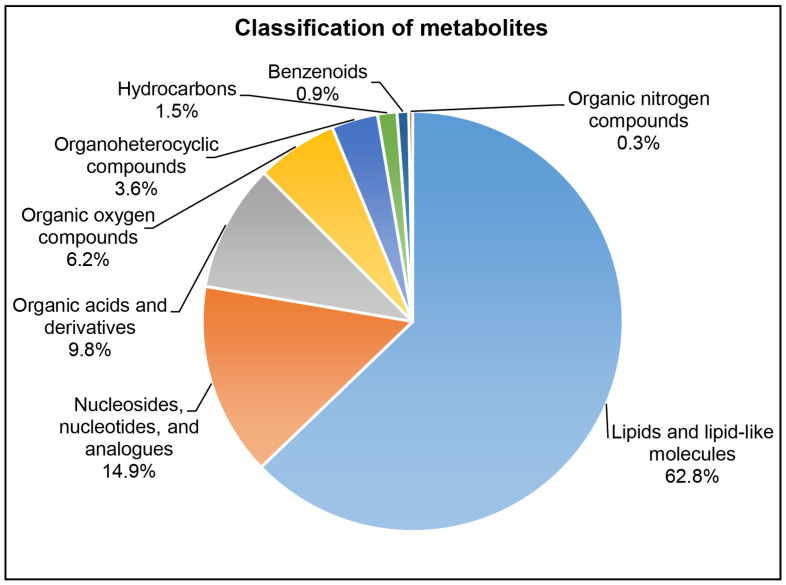
Spermatozoa metabolome classified into chemical super classes with their respective percentages as identified by LC-MS/MS. The classification is based on ClassyFire (https://jcheminf.biomedcentral.com/articles/10.1186/s13321-016-0174-y, accessed on 23 February 2026) and Human Metabolome Database.

For the metabolomic analysis, a total of 469 identified features (peak intensities) were processed in spermatozoa across the three age groups. Annotation by HMDB identified 380 metabolites, whereas 89 peak intensities were not identified. Among these 380 metabolites, the major metabolite components of spermatozoa were lipids and lipid-like molecules; nucleosides, nucleotides, and analogues; organic acids and derivatives; and organic oxygen compounds (Figure 1). A nearly undetectable L-homocysteine was found in the spermatozoa of all the replicates of the advanced age group compared to the young adult group. Conversely, the abundance of methyloctadecanoyl-CoA was nearly undetectable in spermatozoa of all the replicates of the young adult and late adult groups compared to the advanced age group.

**Figure 2:**
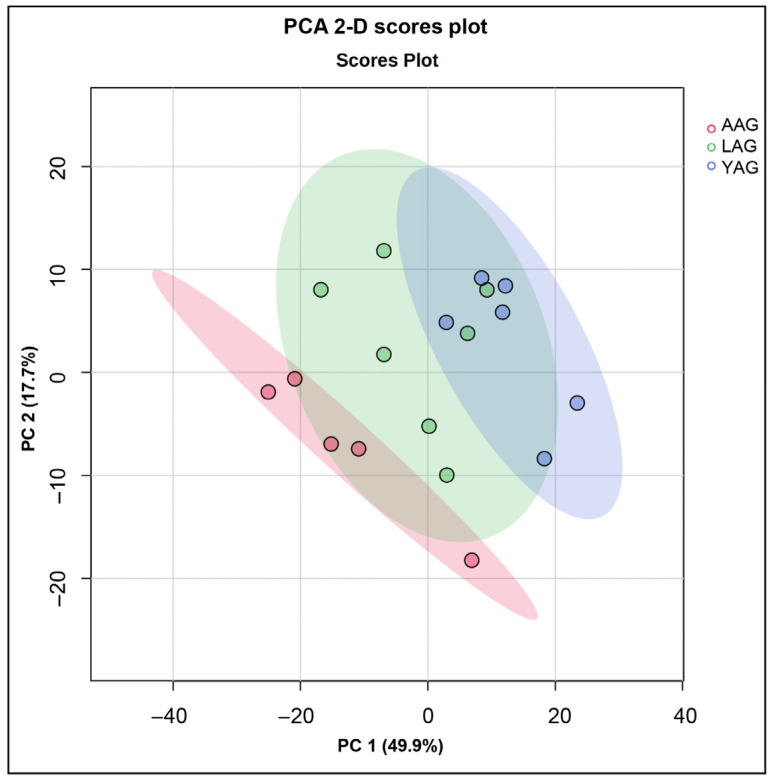
The principal component analysis (PCA) of sperm metabolome during aging. Age groups of men are young adult group (YAG, 21–30 years; *n* = 6), late adult group (LAG, 31–40 years; *n* = 7), and advanced age group (AAG, 41–51 years; *n* = 5). The 2-D PCA scores plot shows that the metabolite data for YAG and AAG separated into two distinct separate clusters, whereas the metabolite data for the LAG showed transitional alignment with the two groups.

**Figure 3:**
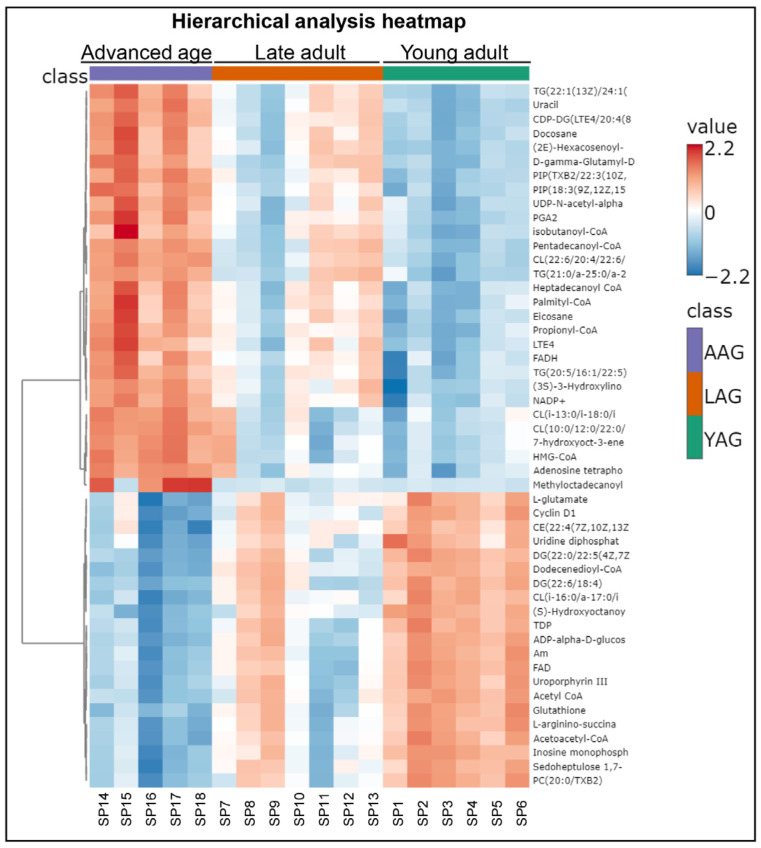
Heatmap for abundance of sperm metabolome during aging. Age groups of men are young adult group (YAG, 21–30 years; *n* = 6), late adult group (LAG, 31–40 years; *n* = 7), and advanced age group (AAG, 41–51 years; *n* = 5). The figure shows only the top 50 differentially significant (*p* < 0.05) metabolites (for figure clarity) ranked according to *p*-values; red color = upregulated metabolites, blue color = downregulated metabolites, and SP = sperm samples.

Initial unsupervised principal component analysis (PCA) resolved the metabolome abundance of data for the young adult and advanced age groups into two distinct separate clusters, indicating significant differential alteration between the two age groups with no intersection of 95% confidence interval ellipses (Figure 2). The abundance data for the late adult group showed a transitional alignment with both the clusters. The first two principal components (PC1, PC2) accounted for the majority of variance (49.9%, 17.7%, respectively) in the metabolomic dataset. The hierarchical clustering and heatmaps of altered metabolites (Figure 3) showed a sharp differentiation of upregulated (red) and downregulated (blue) metabolites in the advanced age group versus the young adult group, whereas the metabolite abundance in the late adult group did not have a clear demarcation and aligned with both the other groups.

The analysis of variance (ANOVA) revealed significant (*p* < 0.05) differences in 164 of 380 metabolites among the three age groups (Supplementary Figure S1; Supplementary Table S1).

The analyses for Spearman correlation, Pattern Hunter, graphical model of debiased sparse partial correlation (DSPC) network and significance analysis of microarray (SAM) are presented in Supplementary Figure S2. The Spearman rank correlation analysis illustrated by the heatmap of 380 showed significant positive and negative correlations among metabolites and clustered them into few correlated groups. Pattern Hunter analysis for correlations of progressive changes with advancing age showed 206 metabolites (113 positive and 93 negative) with significant (*p* < 0.05) progressive correlations (Supplementary Table S2). Graphical model of DSPC network analysis showed that the sperm metabolites having stronger associations clustered together. The SAM analysis identified 80 significant (*p* < 0.05) differentially abundant metabolites (Supplementary Table S3).

**Figure 4:**
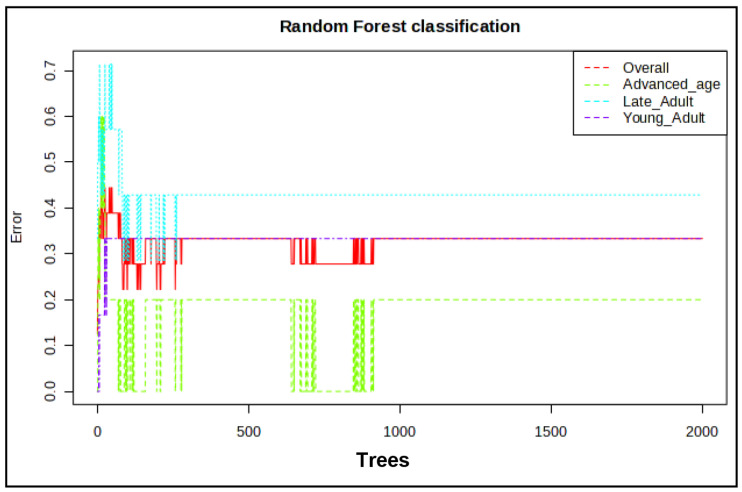
Random forest regression analysis of sperm metabolome during aging. Age groups of men are young adult group (21–30 years; *n* = 6), late adult group (31–40 years; *n* = 7), and advanced age group (41–51 years; *n* = 5). The figure shows random forest classification model with cumulative error rates measured for each group.

The partial least squares-discriminant analysis (PLS-DA) further showed that the young adult group was distinct from the advanced age group, whereas the late adult group aligned intermediately with young and advanced age groups (Supplementary Figure S3). The PC1 and PC2 accounted for 48.2% and 18.4%, respectively, of variance and cross-validated the model based on the original class assignment. The permutation was significant (*p* < 0.006), indicating the validity of the model. The variable importance in projection (VIP) scores for all metabolites ranged from 0.004 to 1.5787 using PC1. A total of 171 metabolites exceeded the score of 1 for all the analyzed components (Supplementary Table S4). The sparse (s) PLS-DA further validated the model and showed the clear separation of the metabolite data between the young adult and advanced age groups. The cumulative error rates from the random forest (RF) model analysis of the three age groups also showed differences between the young adult and advanced age groups (Figure 4).

#### 2.2.2. Pairwise Group Analysis

**Figure 5:**
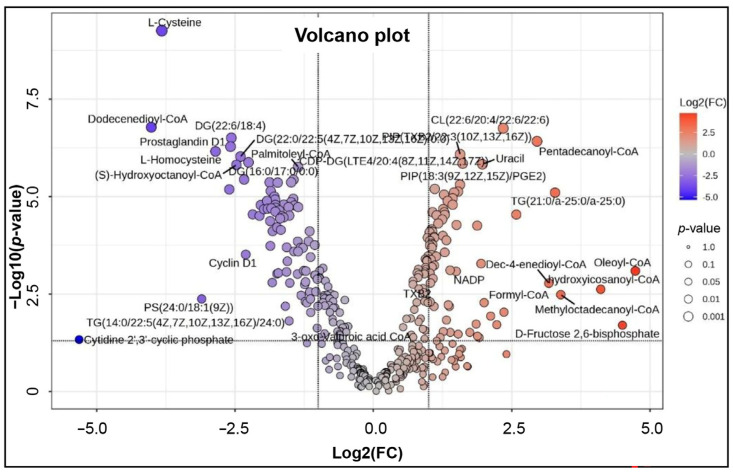
Pairwise statistical analysis of sperm metabolome during aging. Age groups of men are young adult group (21–30 years; *n* = 6) and advanced age group (41–51 years; *n* = 5). The figure shows volcano plot for 153 significant metabolites (threshold of *p* < 0.05 and FC ≥ 2).

**Figure 6:**
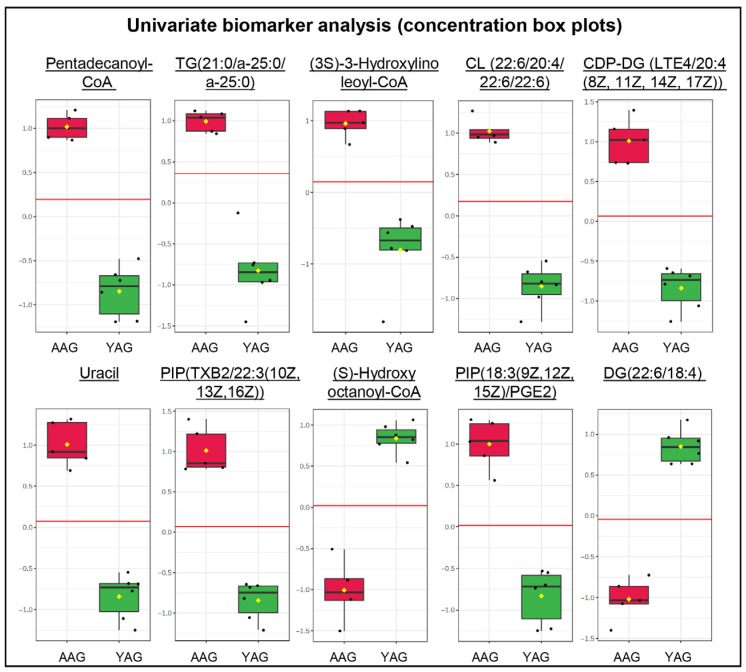
The univariate biomarker analysis of sperm metabolome during aging. Age groups of men are young adult group (YAG, 21–30 years; *n* = 6) and advanced age group (AAG, 41–51 years; *n* = 5). The figure shows box plots for the top 10 biomarker metabolites: pentadecanoyl-CoA; triacylglycerol (TG)(21:0/a-25:0/a-25:0); (3S)-3-hydroxylinoleoyl-CoA; cardiolipin (CL)(22:6/20:4/22:6/22:6); cytidine diphosphate-diacylglycerol (CDP-DG) (LTE4/20:4(8Z,11Z,14Z,17Z)); uracil; phosphatidylinositol phosphate (PIP) (TXB2/22:3(10Z,13Z,16Z)); (S)-hydroxyoctanoyl-CoA; phosphatidylinositol phosphate (PIP) (18:3(9Z,12Z,15Z)/PGE2)); and diacylglycerol (DG) (22:6/18:4). These were identified as biomarkers based on ROC analysis with AUC = 1. The box plots reveal a clear distinction in biomarker concentrations between YAG and AAG groups. ROC = receiver operating characteristic curve; AUC = area under curve.

Pairwise comparison of the data for the young adult group and advanced age group by volcano plot analysis (combining the significance threshold of *p* < 0.05 and fold change of ≥2) revealed 153 of 380 significantly altered metabolites between the two groups (Figure 5; Supplementary Table S5). Of these 82 metabolites were downregulated and 71 metabolites upregulated. These differentially altered metabolites between the two groups were profiled by heatmaps (Supplementary Figure S4). The PCA analysis again confirmed the data for the young adult group and advanced age group resolving into two separate clusters (Supplementary Figure S5). Further modeling by PLS-DA (Supplementary Figure S6) and sPLS-DA further validated the model and confirmed the clear separation of the metabolite data between the young adult and advanced age groups. Univariate biomarker analysis revealed a total of 197 metabolites with a receiver operating characteristic curve (ROC) area under curve (AUC) score of >0.9, of which 137 showed a perfect ROC score of 1. The top 25 metabolites with an ROC value of 1 are shown in Table 2, whereas the complete list of ROC values for all 380 metabolites is given in Supplementary Table S6. Table 2 shows the top 25 differentially expressed metabolites with discriminatory performance on ROC analysis (AUC = 1.0), indicating robust separation between the young adult group and advanced age group. These metabolites spanned multiple chemical classes, including lipids (triacylglycerols, diacylglycerols, phospholipids, cardiolipins), fatty acyl-CoA derivatives, nucleotide-related metabolites, redox cofactors, and amino acid-related compounds. Several metabolites showed marked upregulation, while others were strongly downregulated. The box and whisker plots for the top 10 metabolites reveal a clear difference in the abundance of the 10 metabolites between the young adult group and advanced age group (Figure 6). Among these top 10 metabolites, pentadecanoyl-CoA, TG(21:0/a-25:0/a-25:0, (3S)-3-hydroxylinoleoyl-CoA, CL(22:6/20:4/22:6/22:6), CDP-DG(LTE4/20:4(8Z,11Z,14Z,17Z)), uracil, PIP(TXB2/22:3(10Z,13Z,16Z)), and PIP(18:3(9Z,12Z,15Z)/PGE2)) showed higher abundance level in the advanced-age group, whereas (S)-hydroxyoctanoyl-CoA and DG(22:6/18:4) were markedly reduced.

**Figure 7:**
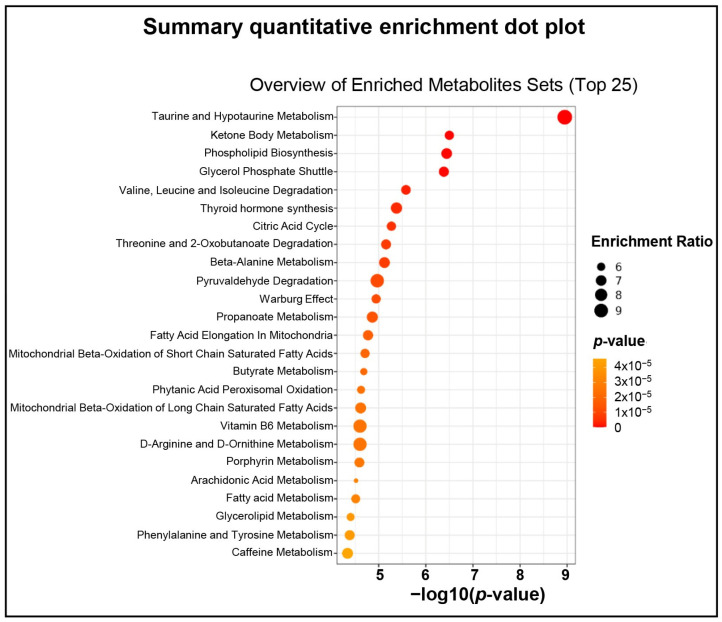
Summary quantitative enrichment analysis (QEA) dot blot of sperm metabolome data during aging. Age groups of men are young adult group (21–30 years; *n* = 6) and advanced age group (41–51 years; *n* = 5). The QEA revealed 97 key metabolic processes that were significantly (*p* < 0.05) enriched (only top 25 shown for figure clarity).

**Figure 8:**
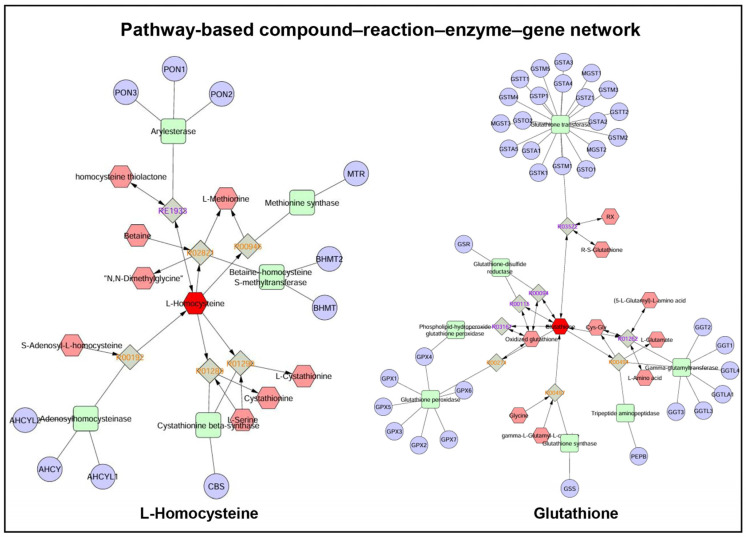
Pathway-based compound–reaction–enzyme–gene network for L-homocysteine and glutathione. L-homocysteine can influence the redox balance by modulating glutathione synthesis. Glutathione is critical for neutralizing reactive oxygen species. Disruptions in this network can exacerbate oxidative damage, thereby affecting sperm function and integrity. L-homocysteine and glutathione are shown as dark red hexagons. Grey squares represent reaction nodes showing reaction IDs; light red hexagons represent composite nodes; green squares represent enzyme nodes; blue circles represent gene nodes; and arrows indicate reaction direction, with single-headed arrows denoting unidirectional reactions (orange reaction IDs), whereas double-headed arrows indicating bidirectional (reversible) reactions (purple reaction IDs).

Quantitative enrichment analysis revealed 97 key significantly enriched metabolic processes (Figure 7; Supplementary Table S7). These enriched pathways included crucial metabolic pathways, including taurine and hypotaurine metabolism, ketone body metabolism, phospholipid biosynthesis, citric acid cycle, glycolysis/gluconeogenesis, glutathione metabolism, mitochondrial beta-oxidation of long chain saturated fatty acids, fatty acid metabolism, etc. As an example, the pathway-based compound–reaction–enzyme–gene networks of L-homocysteine and glutathione are illustrated to highlight their interconnected roles in regulating oxidative stress (Figure 8).

The pairwise analysis of metabolome data for the young adult versus late adult groups and late adult versus advanced age groups showed no separate clustering of the groups in both comparisons, with 95% confidence ellipses of the groups intersecting each other (Supplementary Figure S7). The heatmap analysis confirmed the overlap of data, and volcano plot analyses revealed 18 metabolites differing in abundance between the young adult and late adult groups and 25 metabolites differing in abundance between the late adult and advanced age groups (Supplementary Figure S7). These differential metabolites for both comparisons were accounted for in the differential metabolites between the young adult group and advanced age group.

## 3. Discussion

In this study, significant alterations in metabolome abundance were observed in the advanced age group, suggesting potential molecular disruptions that may compromise sperm function with aging. Gross and microscopic semen analysis revealed no significant differences in any endpoints among the three age groups, except a higher sperm concentration in the late adult group versus the young adult group. Previous studies reported a deterioration in semen parameters with age, notably, reduced semen volume by about 35 years [9,12,34] and reduced sperm count, viability, and motility by about 40 years of age [11,12,35,36,37]. Recently, lower sperm concentrations were reported in men aged 21–30 compared to those aged 31–50 but similar to men over 50 [17]. Our results align with these mixed reports.

Metabolomic profiling identified 380 metabolites across three age groups. This dataset encompassed a diverse range of metabolite classes; lipids and lipid-like molecules were most abundant, followed by nucleosides, organic acid derivatives, organic oxygen compounds, and organoheterocyclic compounds, with organic nitrogen compounds least abundant. A similar metabolite distribution has been previously reported in spermatozoa from humans [52,56], bulls [57], and boars [58], underlining their critical roles in structural integrity, energy metabolism, and overall physiological and developmental functions of spermatozoa.

Analyses by unsupervised and supervised statistical tests consistently revealed the distinct separation of sperm metabolome abundances between the young adult group and advanced age group, forming two well-separated clusters. These distinctions were due to significant differences in 164 metabolites across age groups as shown by one-way ANOVA. Notably, L-homocysteine was nearly undetectable in all advanced age samples, contrasting with its high abundance in younger age groups. Conversely, methyloctadecanoyl-CoA was nearly undetectable in the young and late adult groups but abundantly detected in the advanced age-group, indicating distinct age-associated metabolic alterations in spermatozoa.

L-homocysteine, a sulfur-containing amino acid produced during methionine metabolism [59], is crucial in remethylation and transsulfuration pathways, with hyperhomocysteinemia linked to various disorders [60]. More discussion on L-homocysteine is given later in the discussion on altered organic acid metabolites. Methyloctadecanoyl-CoA, a long-chain acyl-CoA derived from fatty acids, belongs to a group of critical intermediates in lipid metabolism, serving as substrates for β-oxidation and complex lipid biosynthesis [61]. Acyl-CoAs also regulate cellular processes including post-translational protein acylation and enzyme activity. In this study, methyloctadecanoyl-CoA was abundant in the spermatozoa of advanced age men but nearly undetectable in younger groups. While the significance is unclear, excessive acyl-CoA tissue accumulation was linked to lipotoxicity, and impaired cellular physiology [61], warranting further investigations into its role in sperm function and age-related reproductive decline.

Studies on human aging and sperm metabolomics are not available. However, one study examined the metabolomic profile of whole semen in younger (26–30 years) and older (45–54 years) men seeking fertility consultations [54]. Potential compromised fertility may have confounded the age-related effects in the reported study. The reported study [54] revealed 129 altered metabolites in the older group including elevated oxidized glutathione and reduced uracil. Our results revealed 153 altered metabolites (of a total of 380) in the advanced age group compared to the young adult group and supported decreased glutathione levels, but in contrast, uracil was upregulated in the advanced age group. The reasons for this discrepancy are not known but may be related to the metabolomics of whole semen in the reported study [54] versus the purified spermatozoa in the current study. The upregulation of uracil in the current study is further supported by the concurrent downregulation of CTP, which suggests age-associated disturbances in pyrimidine metabolism, potentially reflecting impaired nucleotide biosynthesis alongside increased RNA turnover or degradation. The four metabolites identified as biomarkers in the reported study [54], namely, pipamperone, 2,2-bis(hydroxymethyl)-2,2′,2″-nitrilotriethanol, arg-pro, and triethyl phosphate, were not identified in our metabolome dataset.

Despite the lack of prior studies specifically addressing aging-related changes in sperm metabolomics, several investigations have provided important insights into the human sperm metabolomic landscape in relation to fertility. Studies in infertile men consistently report metabolic disturbances characterized by increased monounsaturated and saturated fatty acids (e.g., stearic, palmitic, myristic) and reduced polyunsaturated fatty acids, including docosahexaenoic, arachidonic, linoleic, and linolenic acids, in asthenozoospermic spermatozoa [47,48]. Similar elevations in fatty acids such as palmitic, stearic, oleic, linoleic, arachidonic, and docosahexaenoic acids have also been observed in asthenozoospermic, asthenoteratozoospermic, and oligoasthenoteratozoospermic men [49], suggesting impaired membrane fluidity and motility. Metabolomic alterations have been identified in idiopathic asthenozoospermia, including reduced levels of amino acids (tryptophan, glutamic acid, leucine, cysteine) and nucleotide components (guanosine, cytidine), indicating compromised metabolic, antioxidant, and genetic functions [50]. Another study further revealed reduced lysophosphatidylserine 18:1, diacylglycerols 32:0, and triacylglycerols 48:1, alongside increased cardiolipins and cholesterol in asthenozoospermic spermatozoa [51]. LC-MS profiling also identified disruptions in amino acid, energy, lipid metabolism, and redox balance, with decreased methionine, isoleucine, and arginine and elevated galactose, fructose-1-phosphate, dihydroxyacetone-phosphate, and glycerol-3-phosphate in asthenozoospermic men [52]. In obesity-associated asthenozoospermia, additional metabolic stress was evident, marked by altered glutamate, proline, and glutathione pathways [45]. A lipidomic analysis of sperm membranes in infertile sperm identified reduced seminolipid, phosphatidic acid, phosphatidylcholine, and lysophosphatidylethanolamine and elevated cholesterol sulfate [53]. These studies demonstrate complex metabolomic imbalances closely linked to impaired sperm motility and male infertility.

In the present aging study, biomarker analysis identified 137 metabolites with an AUC of 1, indicating their potential as robust biomarkers for advanced sperm aging. Accordingly, this discussion focuses on key potential biomarker metabolites as follows.

Several saturated fatty acyl-CoAs were dysregulated in the spermatozoa of advanced age men. Pentadecenoyl-CoA, propionyl-CoA, HMG-CoA, and hydroxyicosanoyl-CoA were upregulated, whereas (S)-hydroxyoctanoyl-CoA, (3R)-3-hydroxyoctanoyl-CoA, and malonyl-CoA semialdehyde were downregulated. The role of pentadecanoyl-CoA in aging remains unclear. Upregulation of propionyl-CoA may contribute to impaired sperm energy metabolism and potentially affect ATP availability and sperm motility [62], while increased HMG-CoA may disrupt cholesterol biosynthesis and membrane remodeling, essential for sperm capacitation [63]. Upregulated hydroxyicosanoyl-CoA may reflect a shift toward altered lipid and intermediary metabolism, potentially disrupting mitochondrial energy production and compromising sperm motility [64]. The downregulation of (S)-3-Hydroxyoctanoyl-CoA, which is formed from coenzyme A and (S)-3-hydroxyoctanoic acid [65], (3R)-3-hydroxyoctanoyl-CoA and malonyl-CoA semialdehyde suggests compromised short-chain fatty acid metabolism, which may impair mitochondrial efficiency, but their role in aging sperm requires further study.

Several monounsaturated fatty acyl-CoAs, including (2E)-hexacosenoyl-CoA, oleoyl-CoA, and 7-hydroxyoct-3-enedioyl-CoA were upregulated in the advanced age spermatozoa, while palmitoleyl-CoA was downregulated. Dysregulated monounsaturated fatty acids are associated with poor semen quality, disruptions in sperm membrane lipid metabolism, and impaired membrane integrity and function in asthenozoospermic, asthenoteratospermic, and oligoasthenoteratospermic men [48,49]. Downregulation of palmitoleyl-CoA may impact cholesterol homeostasis and sperm membrane dynamics causing sperm capacitation-associated signaling problems [66].

The significance of the upregulation of polyunsaturated fatty acyl-CoA (3S)-3-hydroxylinoleoyl-CoA and the downregulation of ethyl arachidonate in advanced-age spermatozoa need further study. However, dysregulation of polyunsaturated fatty acid (PUFA) metabolites may compromise sperm membrane integrity, redox balance, and functional competence resulting in poor sperm motility, as reported across multiple abnormal semen phenotypes [47,49].

Several cardiolipins (CLs) were altered in advanced age spermatozoa including CL(22:6/20:4/22:6/22:6), which was upregulated, and CL(i-16:0/a-17:0/i-17:0/20:0) and CL(14:0/14:0/18:4(6Z,9Z,12Z,15Z)/18:2(9Z,12Z)), which were downregulated. The specific functional roles of each of these CL species in aging spermatozoa remain unclear; however, CLs are key phospholipids of the inner mitochondrial membrane essential for mitochondrial biogenesis, respiration, and protein import, and their dysregulation may reflect impaired mitochondrial integrity and bioenergetic efficiency of spermatozoa [51,67,68].

Several triacylglycerols (TGs) were altered in advanced age spermatozoa including TG(21:0/a-25:0/a-25:0) and TG(22:1(13Z)/24:1(15Z)/24:1(15Z)), which were upregulated, and TG(14:0/20:3(5Z,8Z,11Z)/16:1(9Z)), which was downregulated. The specific role of the individual indicated TG species in age-related sperm function remains unclear and requires further study. However, TGs regulate lipid storage and transport [69]. Their upregulation may reduce the availability of free fatty acids required for membrane remodeling and energy production in sperm, whereas downregulation of TGs has been associated with asthenozoospermia, aging, insulin resistance, and infertility [51,70,71].

Several diacylglycerols (DGs) were altered in advanced age spermatozoa including CDP-DG(LTE4/20:4(8Z,11Z,14Z,17Z)), which was upregulated, and DG(22:6/18:4), which was downregulated. A reduced abundance of DG species may compromise acrosome responsiveness, membrane fusion capacity, and sperm–oocyte interaction [72]. Reduced DG levels have been reported in idiopathic infertile bulls [73]. Upregulated CDP-DG may cause age-related sperm dysfunction via oxidative and inflammatory mechanisms, impairing sperm viability and functional competence [65,74].

Several phosphatidylserines (PSs) were altered in advanced age spermatozoa including PS(16:0/15:0) and PS(24:0/18:1(9Z)), which were downregulated. The specific significance of these metabolites in sperm function is not known. PSs are critical components of the sperm plasma membrane, contributing to membrane integrity, apoptosis, capacitation, and sperm–egg fusion [75,76]. Reduced PS levels may compromise membrane stability and affect membrane dynamics, capacitation efficiency, and sperm–oocyte interaction in aging spermatozoa and have been linked to male infertility [77,78].

Phosphatidylinositol phosphate (PIP) metabolites, PIP(TXB2/22:3(10Z,13Z,16Z)) and PIP(18:3(9Z,12Z,15Z)/PGE2)), were upregulated in the advanced age spermatozoa. PIPs regulate oxidative stress, cellular aging, spermatogenesis, and sperm–egg fusion, supporting fertility and sperm maturation [79,80,81]. Upregulation of PIPs may disrupt calcium signaling, membrane remodeling, and fusion events in sperm, required for successful fertilization.

Phosphatidylcholines (PCs) metabolite PC(20:0/TXB2) was downregulated in the advanced age spermatozoa. Reduced levels of PC(20:0/TXB2) in advanced age spermatozoa may compromise membrane fluidity and calcium signaling, thereby impairing sperm motility and acrosome reaction [82,83]. Reduced PC levels were reported in the spermatozoa of men with oligozoospermia and idiopathic infertility [84].

The sphingolipid metabolite, ganglioside GD3, was downregulated in the spermatozoa of the advanced age group, which may disrupt membrane organization and sphingolipid-dependent signaling, potentially impairing capacitation and sperm–egg interaction [85].

The prostaglandin (PG) metabolite, PGD1, was downregulated in the spermatozoa of the advanced age group. Reduced PGD1 may impair motility and capacitation. Reduced levels of several PGs in semen were associated with asthenozoospermia in men [86].

Several nucleosides and derivatives were significantly altered in advanced-age spermatozoa. Nicotinamide adenine dinucleotide phosphate (NADP+) was upregulated, whereas flavin adenine dinucleotide (FAD), inosine monophosphate (IMP), cyclic guanosine monophosphate (cGMP), ADP-α-D-glucose, and uridine diphosphate (UDP) were downregulated. NADP^+^ regulates the redox balance, mitochondrial function, metabolism, and sperm capacitation [87,88], and its upregulation suggests age-associated remodeling of cellular redox homeostasis under elevated oxidative stress in aging spermatozoa. FAD, essential for mitochondrial redox reactions, supports sperm metabolism and function [89], and its downregulation suggests compromised oxidative metabolism and ATP production, potentially affecting sperm motility and viability. Reduced IMP implies impaired purine metabolism [90], reduced cGMP may indicate impaired capacitation and acrosome reaction [91], and reduced UDP points to impaired sperm maturation [92].

Several organic acids were significantly altered in advanced-age spermatozoa. D-gamma-glutamyl-D-glutamic acid and homocystine were upregulated, whereas L-homocysteine, N-myristoyl serine, L-cysteine, glutathione, L-arginino-succinate, and cyclin D1 were downregulated. The upregulation of homocystine, an oxidative product of L-homocysteine, indicates redox imbalance in aging spermatozoa [93]. Although the biological significance of altered or negligible L-homocysteine levels in advanced-age spermatozoa in this study remains unclear, these changes may be linked to enhanced oxidative stress and mitochondrial dysfunction [94]. In this context, endogenous and exogenous antioxidant-associated metabolites, such as L-carnitine and lycopene, have been reported to improve sperm quality and fertility by reducing oxidative stress and protecting sperm DNA integrity [95,96], indicating the potential relevance of redox-modulating pathways in age-associated sperm dysfunction. The reported study [94] found an overabundance of homocysteine in the spermatozoa of normozoospermic infertile men. As a precursor of cysteine, L-homocysteine is essential for glutathione synthesis, a major intracellular antioxidant [97]. Its depletion may compromise redox homeostasis, heighten oxidative stress, disrupt methylation reactions [98,99], and impair testicular function, as reflected by lower seminal plasma concentrations of L-homocysteine in azoospermic men [100]. N-myristoyl serine has unclear roles in sperm but functions in signaling and metabolic homeostasis, etc. [101]. L-cysteine protected against testicular damage and improved sperm quality in bulls [97,102,103]. Reduced malic acid is associated with poor semen quality [104,105]. Glutathione protects spermatozoa from oxidative damage [54,106]; its depletion impairs motility in infertile men, and supplementation improves sperm quality [107]. An aging-associated decrease in glutathione was also observed in rat testis [108]. Cyclin D1 regulates spermatogenesis in mice [109].

Several organic oxygen compounds were downregulated in advanced-age spermatozoa, including fructose 1,6-bisphosphate, sedoheptulose 1,7-bisphosphate, acetyl-CoA, and cytidine triphosphate (CTP). Reduced fructose 1,6-bisphosphate suggests diminished glycolytic flux in spermatozoa, potentially limiting ATP availability required for flagellar movement and sperm motility [110]. Downregulation of sedoheptulose 1,7-bisphosphate may indicate compromised cellular responses to oxidative stress, increasing the vulnerability to ROS-mediated damage [111]. Downregulation of acetyl-CoA implies impaired oxidative phosphorylation, contributing to lower energy production, compromised mitochondrial function, and reduced sperm viability [112]. Reduced CTP suggests altered nucleotide metabolism, potentially affecting RNA synthesis, membrane phospholipid biosynthesis, and sperm maturation processes [113].

Several heterocyclic compounds were significantly altered in advanced-age spermatozoa. Notably, uracil was upregulated, while dodecenedioyl-CoA, acetoacetyl-CoA, and thiamine diphosphate were downregulated. The upregulation of uracil in spermatozoa may reflect increased RNA degradation, impaired RNA processing, or dysregulated nucleotide salvage pathways, potentially affecting post-transcriptional regulation and protein synthesis required for sperm maturation and function [114]. The upregulation of uracil is further supported by the concurrent downregulation of CTP (discussed above), which suggests age-associated disturbances in pyrimidine metabolism. Such imbalance may adversely affect membrane phospholipid synthesis, RNA-dependent processes, and overall sperm functional competence with advancing age. Downregulation of acetoacetyl-CoA indicates reduced cholesterol biosynthesis, which may impair membrane fluidity, capacitation efficiency, and fertilization competence [63,115]. The decrease in thiamine diphosphate (vitamin B1) points to impaired carbohydrate oxidation and reduced energy efficiency, which may negatively impact sperm motility and viability [116].

Quantitative enrichment analysis revealed significant age-related alterations in several sperm metabolic pathways, including taurine and hypotaurine, ketone body, phospholipid biosynthesis, citric acid cycle, fatty acid, and glutathione metabolism. Taurine and hypotaurine metabolism, crucial for antioxidation [117], motility [118], and fertilization [119], was likely affected by reduced L-cysteine. Ketone body metabolism enhances sperm motility [120] and was affected by the upregulation of succinyl-CoA and the downregulation of acetoacetyl-CoA, potentially impacting energy production. Phospholipid biosynthesis, essential for sperm membrane structure and motility [84], was disrupted through alterations in phospholipids [121]. The citric acid cycle of oxidative phosphorylation and glycolysis provides energy for sperm function [122] and was impacted by age-associated alterations in malic acid, ATP, succinyl-CoA, and FAD. Glutathione metabolism is important for redox balance, sperm energy metabolism, and sperm motility [54,123]. Glutathione, a major intracellular antioxidant, is critical for neutralizing reactive oxygen species. In this regard, L-homocysteine serves as a key intermediate in the methionine cycle and can influence the redox balance by modulating glutathione synthesis. Disruptions in this network can exacerbate oxidative damage, thereby affecting sperm function and integrity. The age-related dysregulation of NADP+, ATP, L-cysteine, and FAD in this pathway may have a potential impact on sperm functions.

In summary, this study identifies age-associated differences in sperm metabolomic profiles that may contribute to alterations in sperm development and function. While robust unsupervised and supervised statistical approaches were applied, the relatively small sample size and the identification of multiple candidate metabolites warrant cautious interpretation. Given the lack of prior studies examining age-related changes in human sperm metabolomics, the present findings provide an initial framework for understanding age-associated alterations in the sperm metabolome. Validation in larger, independent, and well-characterized cohorts that also account for lifestyle, physical activity, and other potential confounders is therefore required to confirm the robustness, biological relevance, and translational potential of the identified metabolites.

## 4. Materials and Methods

### 4.1. Study Design and Sample Collection

The research protocol for this study received approval from the Bioethics Committee at the Center for Excellence in Genomic Medicine and Research, King Abdulaziz University, Jeddah, Saudi Arabia (approval code: 11-CEGMR-Bioeth-2020; Approval date: 18 December 2020). The study design and sample collection have been described in detail in a recent study [55]. Briefly, healthy fertile Arab/Saudi married men aged 21–51 years who had naturally fathered at least one child were recruited after informed consent and comprehensive urological evaluation. Semen samples were collected after 3–5 days of abstinence at the King Fahd Medical Research Center, King Abdulaziz University. Men with reproductive, genetic, systemic, or seminal abnormalities were excluded. Of 24 recruited men, 18 qualified samples were included and categorized into three age groups: 21–30 years (*n* = 6), 31–40 years (*n* = 7), and 41–51 years (*n* = 5), reflecting reproductively active decades and the three highest percentage contributions to fatherhood [24,50,124,125]. The 41–50-year age group is considered advanced paternal age, as several studies have established the male age around 40 years as a critical threshold for the onset of significant declines in semen quality, quantity, and overall fertility potential [9,10,12,34,37].

### 4.2. Semen Analysis, Processing, and Sperm Purification

The semen samples were incubated at 37 °C for 30 min for liquefaction and analyzed by the World Health Organization protocol [126]. Briefly, the gross examination of the semen included assessment of the semen volume, pH, consistency, and color. Microscopic examination was performed using a Makler counting chamber or a slide to determine the sperm concentration, total and progressive motility, total sperm count per ejaculate, total live sperm count per ejaculate, and sperm morphology. The presence of somatic/round cells and sperm agglutination was also evaluated. Following the macro- and micro-examination, the spermatozoa were separated from the seminal plasma by centrifugation at 1000× *g* for 10 min, and the resulting sperm pellet was resuspended in 2 mL of phosphate-buffered saline (PBS). The motile spermatozoa were isolated by using a density gradient centrifugation method by gently layering 1 mL of sperm suspension over two layers of gradient solution with a lower layer of 1 mL of 80% and an upper layer of 1 mL of 40% PureCeption (Cooper Surgical, Ballerup, Denmark) in a 15 mL conical tube, followed by centrifugation at 500× *g* for 10 min. The gradient and dead cell layers were carefully aspirated, and the sperm pellet (approximately 100 µL) was resuspended in 6 mL of PBS. The purified sperm cells were recounted using a Makler counting chamber and centrifuged again (1000× *g* for 10 min) to remove PBS. The final purified sperm pellet was then aliquoted, labelled, and stored at −80 °C until further metabolomic analysis.

### 4.3. Metabolome Analysis

#### 4.3.1. Metabolite Extraction

The extraction of metabolites was performed from 5 million purified sperm cells from each of the 18 sperm samples in three age groups. The microfuge tubes with the purified sperm cells were thawed at room temperature. Each sperm aliquot had a volume of 20 µL of PBS, with sperm counts ranging from 5 to 12 million per aliquot across samples. To adjust the spermatozoa number for metabolomic extraction, the volume corresponding to exactly 5 million spermatozoa was calculated for each sample using the equation C_1_V_1_ = C_2_V_2_. The calculated volume containing 5 million sperm was adjusted to a final volume of 20 µL with ice-cold PBS to ensure uniform processing conditions. Subsequently, 100 µL of ice-cold distilled water was added followed by vortexing for 3 min. The samples were incubated at −20 °C for 5 min and vortexed again for 3 min; then, 400 µL of ice-cold methanol and acetonitrile mixture (1:1 *v*/*v*) were added. Another vortexing step for 3 min was followed by homogenizing to lyse the spermatozoa, ensuring the efficient disruption of sperm cells and the extraction of metabolites. All steps were performed on ice or at 4 °C. The contents were incubated at −20 °C for 24 h followed by centrifuging at 10,000× *g* for 10 min at 4 °C. The supernatant containing the extracted metabolites was carefully transferred to a small bottle. This approach ensured identical sperm input and equivalent processing volumes across samples, thereby minimizing the normalization bias arising from variable sperm counts and extraction efficiency. The procedure was applied uniformly to all samples using identical reagents, volumes, and incubation conditions, supporting the reproducibility and consistency of the metabolite yield. The metabolomic analysis of supernatants was conducted according to the method previously reported [127,128].

#### 4.3.2. Mass Spectrometry-LC-MS/MS

Mass spectrometry analysis by LC-MS/MS was performed using the LTQ XL™ linear ion trap instrument (ThermoFisher Scientific, Waltham, MA, USA). A volume of 10 µL metabolite extract was injected into the HPLC column (hypersail gold column C18 Part No: 25005-104630) at a 0.250 mL/min flow rate. The metabolite extracts were gradient eluted with mobile phase A, consisting of 99.9% acetonitrile and 0.1% formic acid (0.1% *v*/*v*), and mobile phase B, consisting of 0.1% formic acid in 99.9% water (0.1%, *v*/*v*), using a gradient program in which the percentage ratios of flow gradient for solvents A and B were 100:0 at the start, reaching 50:50 in 20 min, holding at this ratio for further 30 min, reaching 75:25 in 10 min, and finally reaching again 100:0 in 10 min, with a total running time of 70 min at a column temperature of 30 °C. For mass spectrometry, the multistage mass spectrometry (MSn) parameters were used, which included full scanning mode ranging from 80 to 2000 *m*/*z* with helium as the buffer gas, nitrogen as the sheath gas, 40 arbitrary units as the flow rate, a capillary temperature of 270 °C, a voltage of 4.0 V, and the spray voltage set at −3.0 kV. Each metabolite extract was run in triplicate. The method has been described previously [127,128].

#### 4.3.3. Spectral Analysis, Statistical Analysis, and Functional Analysis

The semen analysis endpoints for the three agegroups were assessed by one-way ANOVA. For an endpoint showing an overall significant effect (*p* < 0.05) by ANOVA, further resolution of the differences between individual groups were assessed by using Tukey’s test. LC-MS/MS spectral data from metabolomics analysis were processed (raw data conversion, peak detection, peak integration, noise reduction, baseline correction, peak alignment) using XCMS. The detected peaks and their fragmentation patterns were compared against the Human Metabolome Database (HMBD) to identify potential matches using retention times and mass accuracy to further confirm the identity of the metabolites. After annotation of metabolite signatures, the statistical and metabolite enrichment analyses were performed with Metaboanalyst Pro (v6.0; 2025, https://pro.metaboanalyst.ca/). The pathway-based network analysis was conducted using Metscape [129]. For statistical analysis of the metabolome data, the peak intensity data of the metabolites were normalized by median and transformed by log and auto scaling to reduce variations among the samples (Supplementary Figure S8). Statistical analyses were performed on the overall data for the three age groups as well as on pairwise groups comparing the young adult group separately with the late adult group and the advanced age group and the late adult group with the advanced age group. The probability of *p* < 0.05 was used as the significance threshold. The statistical tests for the three groups included one-way ANOVA, correlation analysis, PCA, PLS-DA, sPLS-DA, DSPC, SAM, hierarchical clustering and heatmaps, and RF analysis. As indicated, the data analyses included both unsupervised and supervised methods to initially provide an overview of the data structure while exploring natural clustering trends and outliers and to further improve class discrimination among groups, assess group separation, and to increase the robustness and easy interpretation of the model. The above analyses were repeated for pairwise analysis, and much of the focus was kept on the comparison of the young adult group and advanced age group. Functional analysis was performed on pairwise groups and included pathway analysis, biomarker analysis, network analysis, and enrichment analysis. The ROC curves were generated to analyze the potential of metabolites as biomarkers for differentiation of advanced age with an ROC AUC cutoff value of 0.9.

## 5. Conclusions

This study characterized age-associated alterations in the sperm metabolome of healthy fertile men across young adult, late adult, and advanced-age groups using LC-MS/MS. Of the 380 identified metabolites, 164 were significantly altered in the advanced-age group, with young adult and advanced-age groups forming two well-separated clusters. Distinct age-dependent metabolic signatures included the near depletion of L-homocysteine and marked enrichment of methyloctadecanoyl-CoA. Biomarker analysis identified 137 metabolites with discriminatory potential for aging spermatozoa. The top 10 biomarker candidates were pentadecanoyl-CoA, TG(21:0/a-25:0/a-25:0), (3S)-3-hydroxylinoleoyl-CoA, CL(22:6/20:4/22:6/22:6), CDP-DG(LTE4/20:4(8Z,11Z,14Z,17Z)), uracil, PIP(TXB2/22:3(10Z,13Z,16Z)), (S)-hydroxyoctanoyl-CoA, PIP(18:3(9Z,12Z,15Z)/PGE2)), and DG(22:6/18:4). Overall, aging was associated with multifactorial metabolic disruptions in spermatozoa affecting spermatogenesis, motility, energy metabolism, redox balance, mitochondrial function, and fertility-related processes. These findings provide a framework for validating the translational potential of candidate metabolites to mitigate age-related fertility decline.

## Figures and Tables

**Table 1:** Semen analysis endpoints in the three age groups of men: young adult (21–30 years), late adult (31–40 years), and advanced age (41–51 years).

Endpoints	Young Adult Group (*n* = 6)	Late Adult Group (*n* = 7)	Advanced Age Group (*n* = 5)	*p*-Value
Age (years)	27.8 ± 2.6 ^a^	34.3 ± 2.9 ^b^	47.2 ± 4.2 ^c^	0.001 *
BMI (kg/m^2^)	28.68 ± 3.75	26.33 ± 2.88	25.90 ± 2.05	0.114
Abstinence (days)	4.2 ± 0.48	4.1 ± 2.0	3.6 ± 0.9	0.489
Total volume (mL)	3.33 ± 1.84	2.47 ± 0.80	5.10 ± 2.79	0.141
Sperm concentration (×10^6^/mL)	55.3 ± 25.54 ^a^	85.4 ± 22.06 ^b^	78.0 ± 38.24 ^ab^	0.022 *
Total motility (%)	63.17 ± 17.91	69.86 ± 9.06	67.40 ± 12.88	0.201
Progression (%)	39.83 ± 10.44	45.43 ± 13.79	48.40 ± 12.26	0.217
Total sperm/ejaculate (×10^6^)	194.33 ±18.21	217 ± 11.36	337.90 ± 18.71	0.396
Total motile sperm (×10^6^)	142.05 ± 19.21	153.44 ± 84.93	220.65 ± 115.68	0.443
Normal morphology (%)	4.5 ± 0.55	6.0 ± 3.61	5.4 ± 1.14	0.169

Data are expressed as means (±SD); overall *p* value is from one-way ANOVA; different superscripts (^a,b,c,^*) indicate significant differences (*p* < 0.05) by Tukey’s test. Data were previously reported in Ref. [55].

**Table 2:** The univariate biomarker analysis of metabolome data in spermatozoa from young adult group (21–30 years; *n* = 6) and advanced age group (41–51 years; *n* = 5).

S.No.	HMDB Nomenclature	LSI Nomenclature	AUC	*p*-Value	FC	Clusters
1	Pentadecanoyl-CoA	FA 15:0;CoA	1.0	3.84 × 10^−7^	2.8509	5
2	Triacylglycerols (TG)(21:0/a-25:0/a-25:0)	TG 21:0/a-25:0/a-25:0	1.0	7.92 × 10^−6^	3.1604	5
3	(3S)-3-Hydroxylinoleoyl-CoA	FA 18:2(9Z,12Z);3OH(S);CoA	1.0	5.57 × 10^−5^	1.7732	5
4	Cardiolipin (CL)(22:6/20:4/22:6/22:6)	CL 22:6/20:4/22:6/22:6	1.0	1.78 × 10^−7^	2.227	5
5	Cytidine diphosphate-diacylglycerol (CDP-DG)(LTE4/20:4(8Z,11Z,14Z,17Z))	CDP-DG LTE4/20:4(8Z,11Z,14Z,17Z)	1.0	1.42 × 10^−6^	1.5145	5
6	Uracil	—	1.0	1.49 × 10^−6^	1.8663	5
7	Phosphatidylinositol phosphate (PIP)(TXB2/22:3(10Z,13Z,16Z))	PIP TXB2/22:3(10Z,13Z,16Z)	1.0	8.26 × 10^−7^	1.4625	5
8	(S)-Hydroxyoctanoyl-CoA	FA 8:0;3OH(S);CoA	1.0	1.55 × 10^−6^	−2.5958	2
9	Phosphatidylinositol phosphate (PIP)(18:3(9Z,12Z,15Z)/PGE2))	PIP 18:3(9Z,12Z,15Z)/PGE2	1.0	4.96 × 10^−6^	1.4663	5
10	Diacylglycerol (DG)(22:6/18:4)	DG 22:6/18:4	1.0	3.14 × 10^−7^	−2.6539	2
11	L-Homocysteine	—	1.0	6.96 × 10^−7^	−3.0394	2
12	N-Myristoyl Serine	N-acyl (NA) 14:0/Serine	1.0	3.11 × 10^−5^	−2.1382	2
13	Nicotinamide adenine dinucleotide phosphate (oxidized; NADP+)	—	1.0	5.34 × 10^−5^	1.4011	5
14	Prostaglandin D1	FA 20:3(5Z,8Z,11Z);[8-12cy5;11OH;9oxo];15OH	1.0	5.25 × 10^−7^	−2.6838	2
15	L-Cysteine	—	1.0	5.58 × 10^−10^	−3.9262	2
16	D-gamma-Glutamyl-D-glutamic acid	—	1.0	1.11 × 10^−5^	1.3433	5
17	Triacylglycerols (TG)(22:1(13Z)/24:1(15Z)/24:1(15Z))	TG 22:1(13Z)/24:1(15Z)/24:1(15Z)	1.0	7.83 × 10^−6^	1.3619	5
18	Disialoganglioside 3 (Ganglioside GD3)	NeuAc2Hex2Cer	1.0	0.000427496	0.78761	5
19	Phosphatidylcholine (PC)(20:0/TXB2)	PC 20:0/TXB2	1.0	2.27 × 10^−5^	−1.7804	2
20	Cardiolipin (CL)(i-16:0/a-17:0/i-17:0/20:0)	CL i-16:0/a-17:0/i-17:0/20:0	1.0	2.06 × 10^−5^	−1.9732	2
21	Triacylglycerols (TG)(14:0/20:3(5Z,8Z,11Z)/16:1(9Z))	TG 14:0/20:3/16:1	1.0	2.87 × 10^−5^	−2.2672	2
22	Ethyl arachidonate	FA 20:4(5Z,8Z,11Z,14Z);Et	1.0	1.87 × 10^−5^	−1.6171	2
23	Cytidine 2′,3′-cyclic phosphate	—	1.0	0.046608806	−5.6521	3
24	Dodecenedioyl-CoA	FA 12:1;COOH;CoA	1.0	1.67 × 10^−7^	−4.0716	2
25	Flavin adenine dinucleotide (FAD)	—	1.0	1.01 × 10^−5^	−1.8738	2

The table lists the top 25 metabolites identified as biomarkers based on receiver operating characteristics (ROC) analysis with an area under curve (AUC) of 1. HMDB = Human Metabolome Database; LSI = Lipid Standard Initiative; *p*-Value = probability; FC = fold change.

## Data Availability

Data are contained within the article and Supplementary Materials. The mass spectrometry metabolomic data have been deposited to Metabolomics Workbench and are available at the NIH Common Fund’s National Metabolomics Data Repository (NMDR) website, the Metabolomics Workbench, https://www.metabolomicsworkbench.org with assigned Study ID ST004272. The Project DOI is: http://dx.doi.org/10.21228/M8X27M.

## Supplementary Materials

The following supporting information can be downloaded at https://www.mdpi.com/article/10.3390/ijms27052386/s1.
