# Supplementary material for: Age-Associated Metabolomic Changes in Human Spermatozoa
Source: Int J Mol Sci. 2026 Mar 4;27(5):2386. doi: 10.3390/ijms27052386 (PMC12986010; doi:10.3390/ijms27052386)
Supplement: Supplementary file 1 [file ijms-27-02386-s001.zip › Supplementary Figures File.pdf]

Supplementary Figure S1

Figure S1. Analysis of Variance (ANOVA) of metabolome data for spermatozoa from young adult group (21–30 years; n=6), late adult group (31–40 years; n=7), and advanced age group (41–51 years; n=5). The figure shows significant differences ($P<0.05$) by ANOVA in 164 of 380 metabolites (colored yellow, orange and red based on the increasing level of significance) of the spermatozoa among the three age groups.

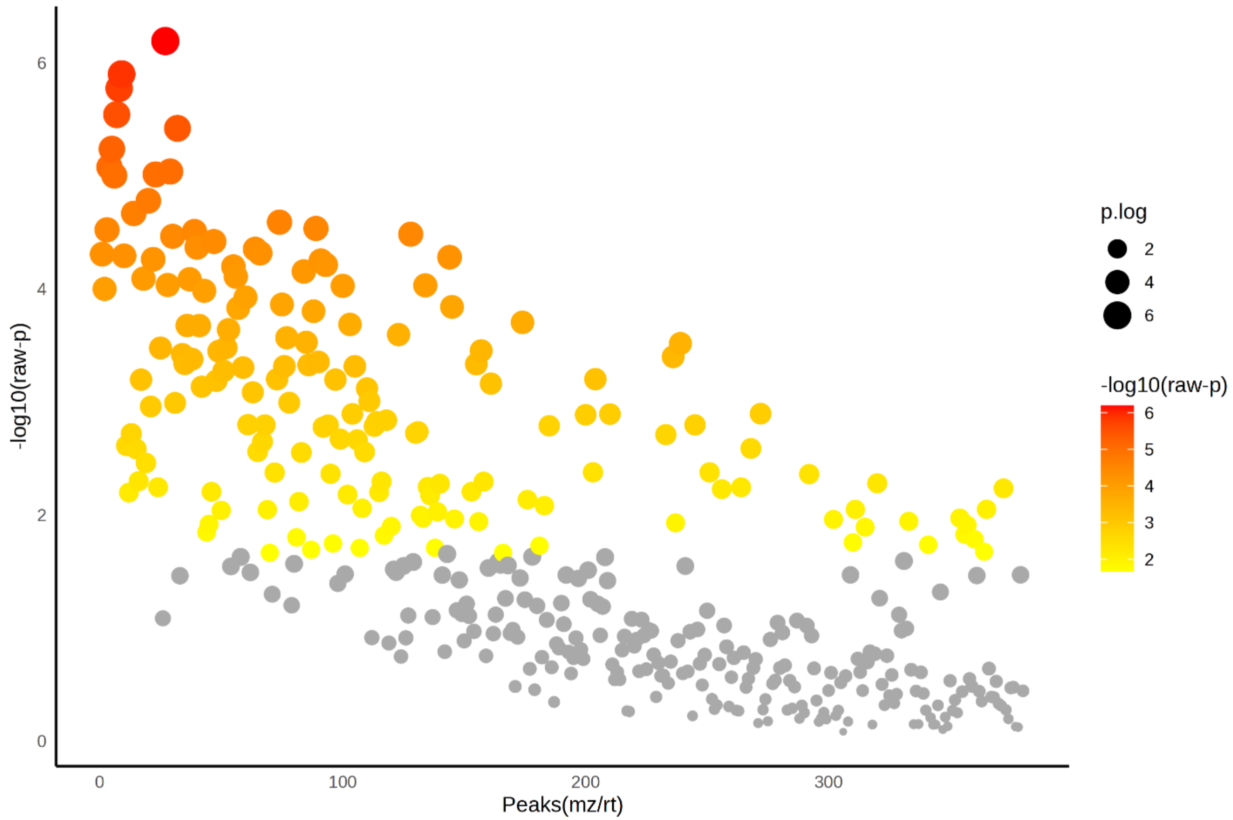

ANOVA analysis plot

Supplementary Figure S2

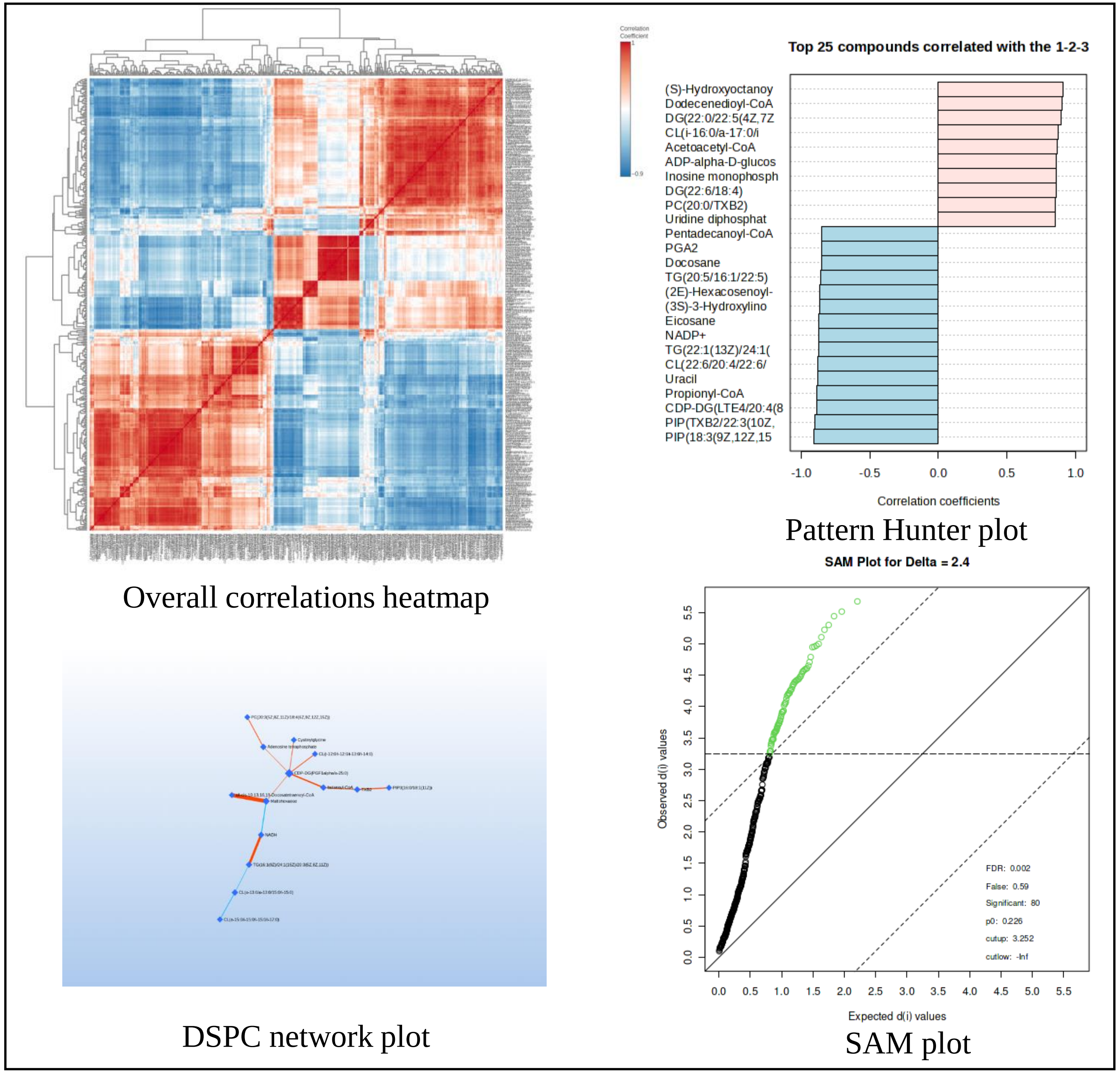

Figure S2. Correlation analysis of metabolome data for spermatozoa from young adult group (21–30 years; n=6), late adult group (31–40 years; n=7), and advanced age group (41–51 years; n=5). The figure shows overall heatmap and hierarchical clustering; Pattern Hunter plot; DSPC network analysis plot; and SAM analysis plot. The 1-2-3 in Pattern Hunter figure indicates pattern of progression in groups.

Supplementary Figure S3

Figure S3. Multivariate regression analysis using Partial Least Squares-Discriminate model (PLS-DA) for the metabolome data in spermatozoa from young adult group (YAG, 21–30 years; n=6), late adult group (LAG, 31–40 years; n=7), and advanced age group (AAG, 41–51 years; n=5). The figure shows PLS-DA pairwise scores plot; PLS-DA 2-D scores plot; loading plot; VIP score plot; PLS-DA classification performance; and permutation test.

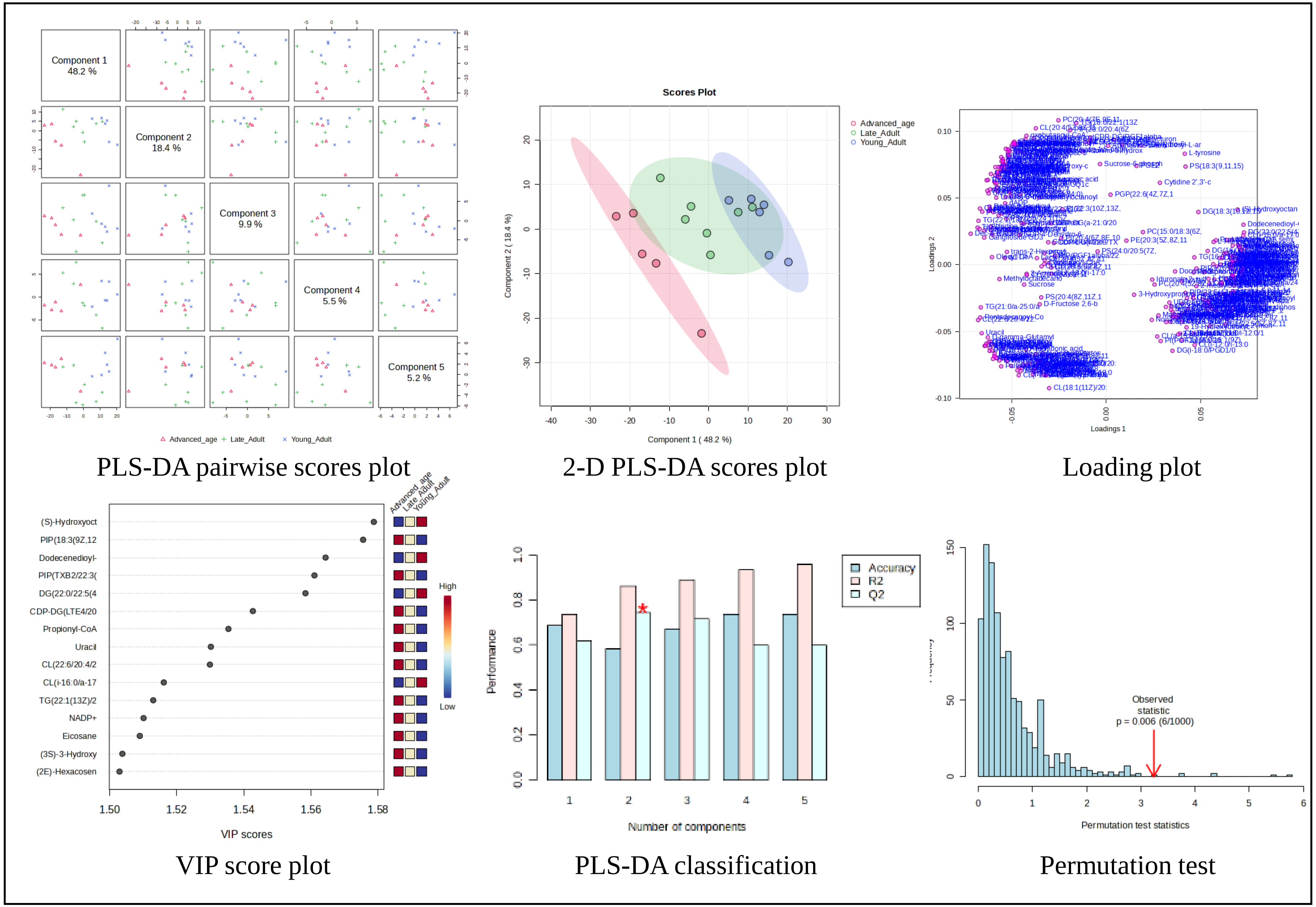

Supplementary Figure S4

Figure S4. Heatmap for abundance of metabolome data for spermatozoa from young adult group (YAG, 21–30 years; n=6) and advanced age group (AAG, 41–51 years; n=5). The figure shows only the top 50 metabolites (for figure clarity). The red color indicates upregulated metabolites, and blue color indicates downregulated metabolites in the AAG compared to YAG.

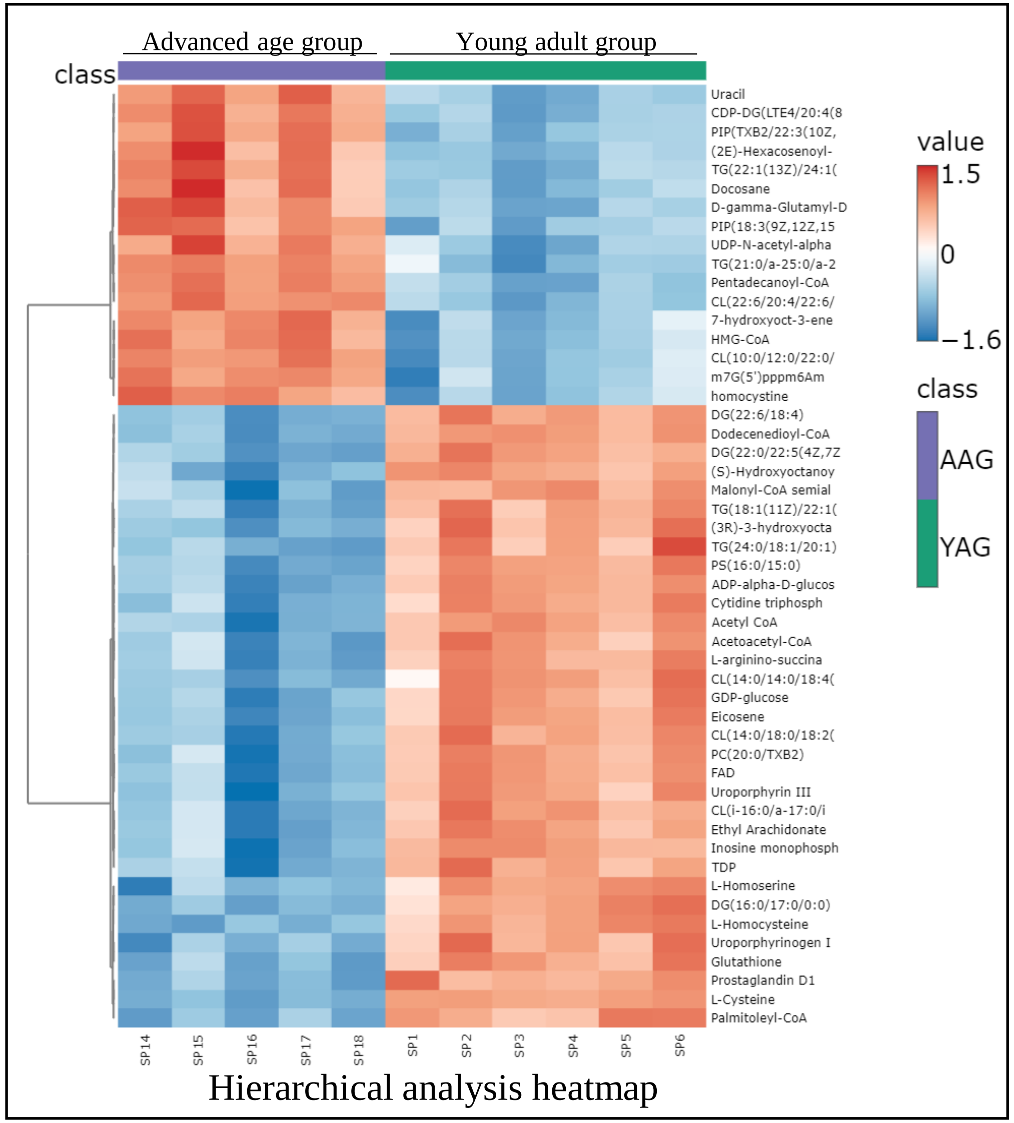

Supplementary Figure S5

Figure S5. Principal component analysis (PCA) of metabolome data for spermatozoa from young adult group (YAG, 21–30 years; n=6) and advanced age group (AAG, 41–51 years; n=5). The figure shows PCA pairwise scores plot; PCA 2-D scores plot; and PCA scree plot.

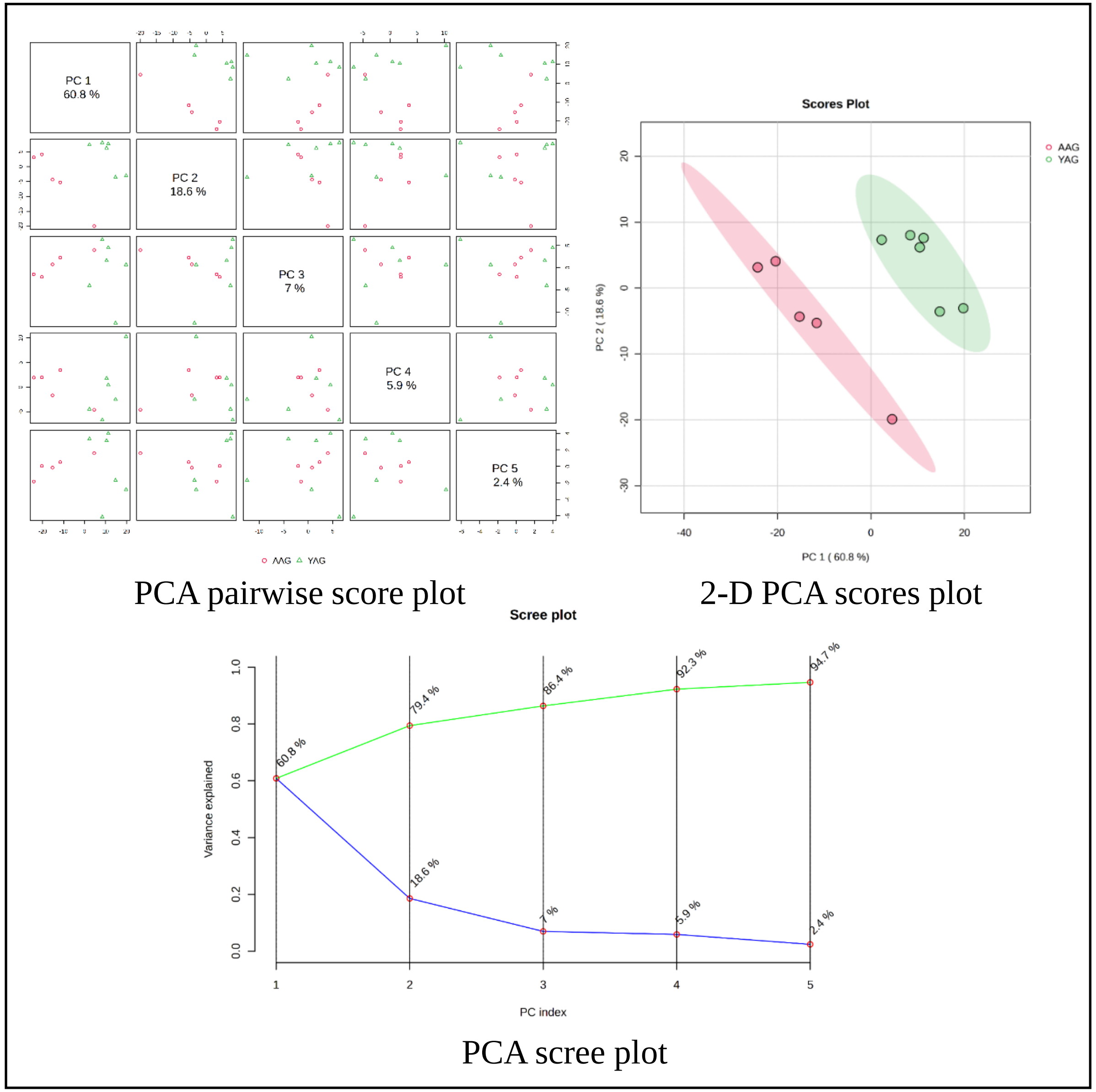

Supplementary Figure S6

Figure S6. Multivariate regression analysis using Partial Least Squares-Discriminate model (PLS-DA) for the metabolome data in spermatozoa from young adult group (YAG, 21–30 years; n=6) and advanced age group (AAG, 41–51 years; n=5). The figure shows PLS-DA pairwise scores plot; PLS-DA 2-D scores plot; loading plot; variable importance in projection (VIP) score plot; and PLS-DA classification performance.

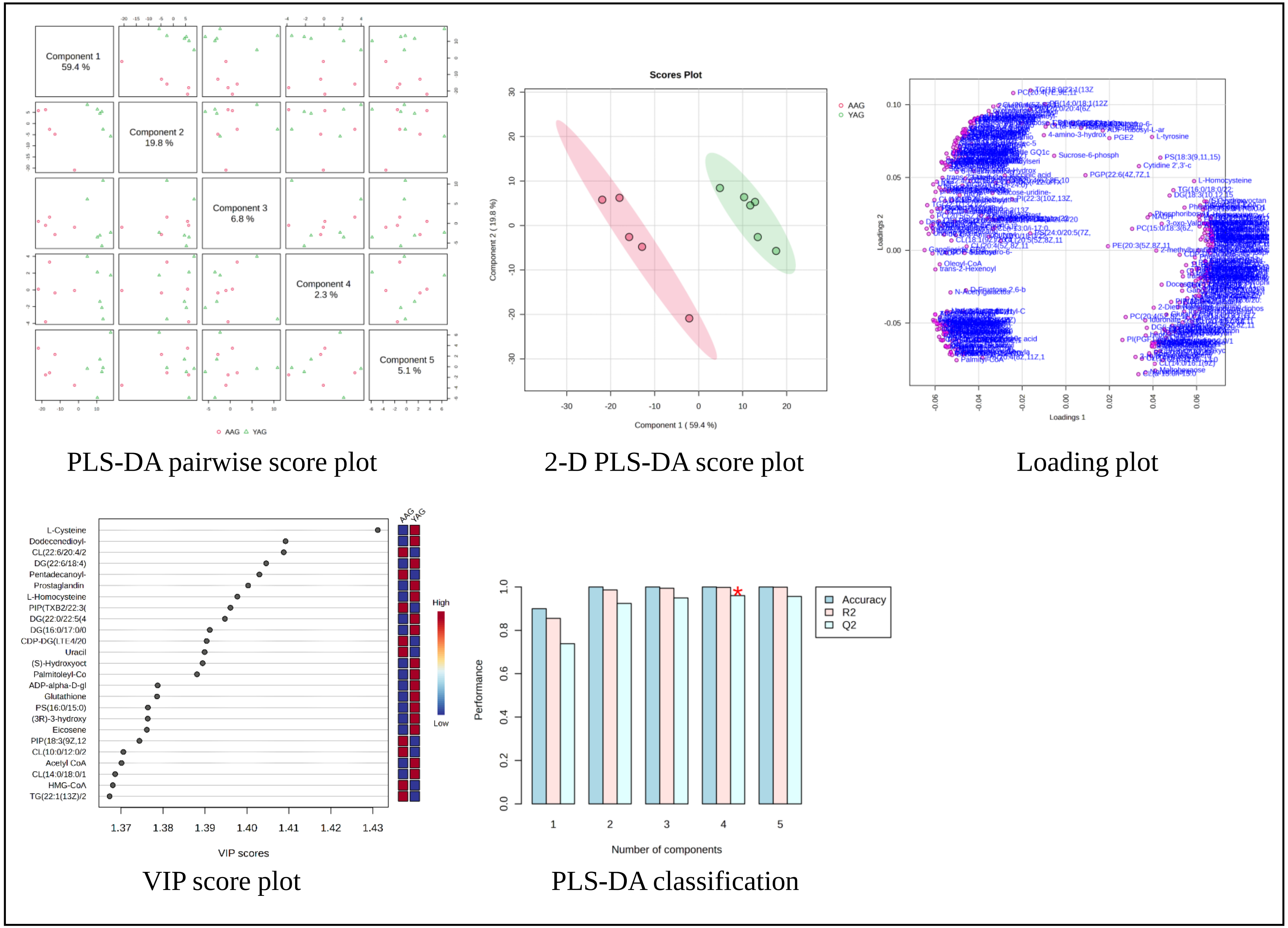

Supplementary Figure S7

Figure S7. The principal component analysis (PCA) and volcano plot of metabolome data of spermatozoa for two pairwise comparisons: left panel; young adult group (YAG, 21–30 years; n=6) versus late adult group (LAG, 31–40 years; n=7) and right panel; late adult group (LAG, 31–40 years; n=7) versus advanced age group (AAG, 41–51 years; n=5). . The figure shows PCA 2-D scores plot; and volcano plot (significant at the threshold of P<0.05 and fold change ≥2).

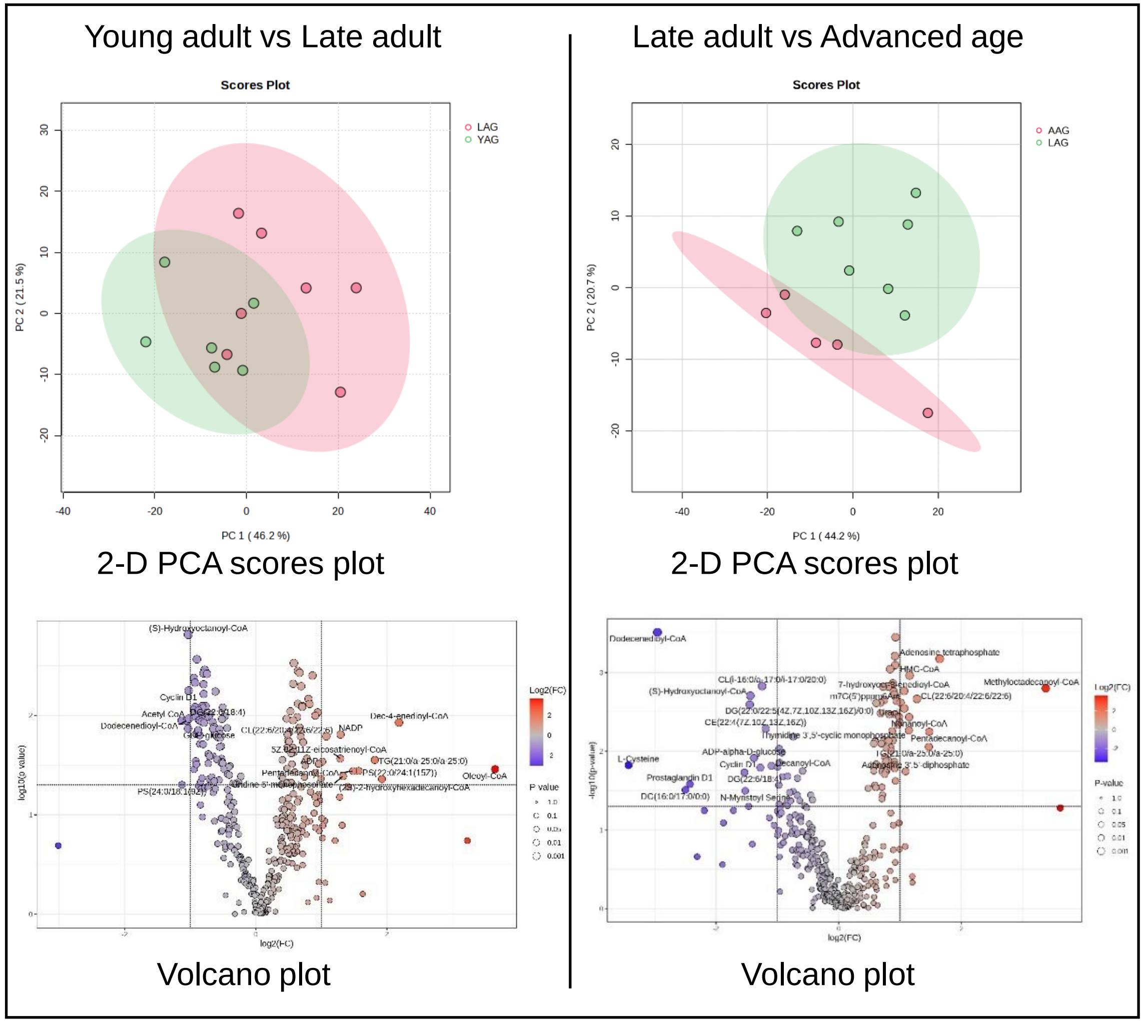

Supplementary Figure S8

Figure S8. Data distribution and normalization of metabolome data for spermatozoa from young adult group (21–30 years; n=6), late adult group (31–40 years; n=7), and advanced age group (41–51 years; n=5). Data for the total 380 metabolites was normalized to median, log transformation, and auto-scaling, and the distribution of peak intensities is shown before and after normalization.

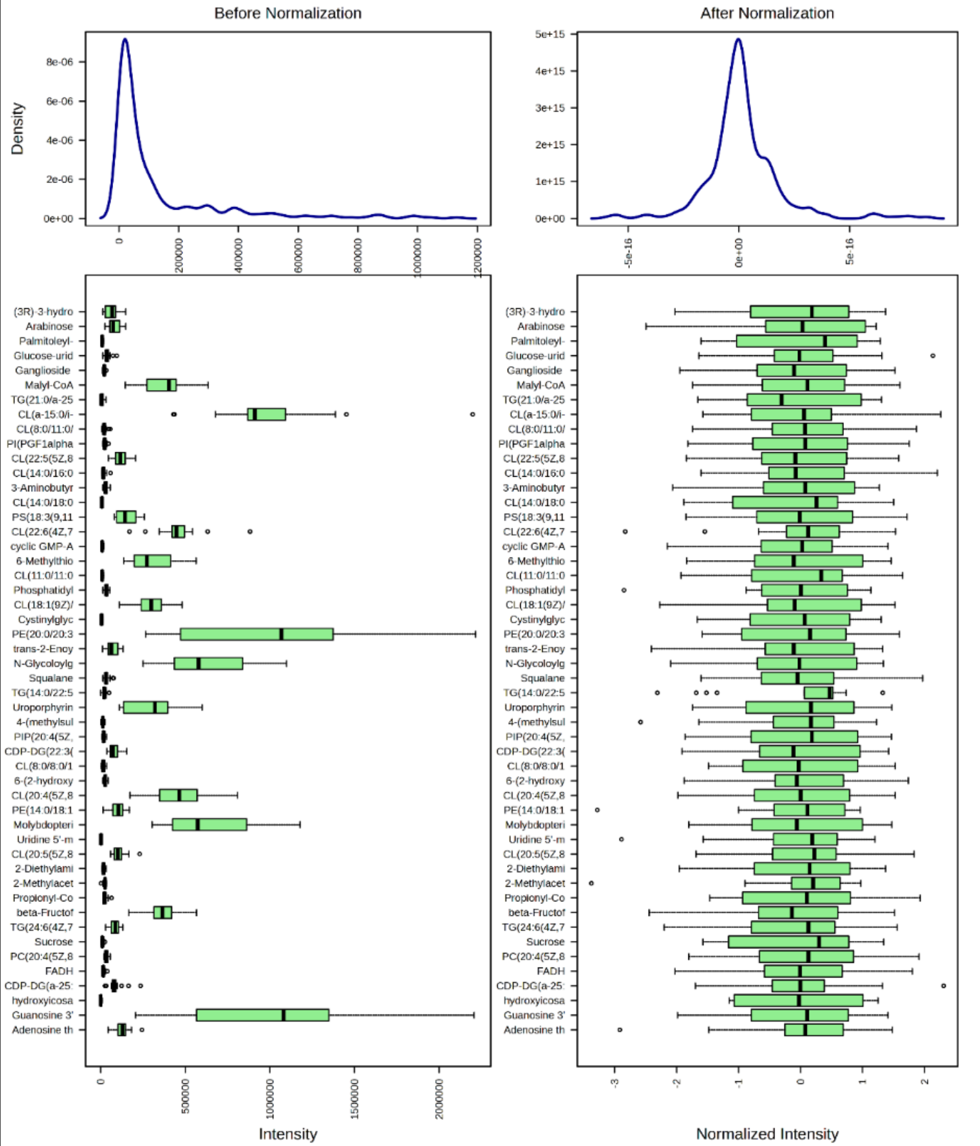

Metabolome data normalization
